# Facet Joint Gouty Arthropathy: An Uncommon Cause of Chronic Lumbar Pain

**DOI:** 10.7759/cureus.93327

**Published:** 2025-09-27

**Authors:** Cara C Chua, Stephanie Ong, Mandy Zhang

**Affiliations:** 1 Sports and Exercise Medicine, Changi General Hospital/SingHealth, Singapore, SGP

**Keywords:** facet joint gouty arthropathy, gout, gouty tophi, lower back pain, spinal gout, tophi

## Abstract

Gout is a well-recognised metabolic disorder typically affecting peripheral joints; however, axial involvement remains underdiagnosed and poorly understood. Facet joint gouty tophi, although rare, represent an important manifestation of gout that can significantly impact patient outcomes. This case report details a 27-year-old gentleman who presented with left-sided lower back pain for the past three years on a background of poorly controlled gout and obesity. A contrast-enhanced MRI revealed T1-weighted contrast enhancement at the L4-L5 facet joint area and a possible punched-out erosion, suggestive of facet joint gouty arthropathy and tophaceous changes, given his history of poorly controlled gout. This case report discusses the prevalence, presentation, imaging and management of facet joint gout arthropathy as an uncommon cause of chronic lower back pain.

## Introduction

Gout is a metabolic disorder caused by the deposition of monosodium urate crystals in joints and tissues, leading to inflammation and damage. The global prevalence of gout is estimated to be around 1-4% [1], with an increasing trend linked to rising obesity and metabolic syndrome. This pattern is also observed in Southeast Asia, including Singapore [2,3].

Gout primarily affects peripheral joints, with the first metatarsophalangeal (MTP) joint being the most common initial site of involvement [2]. Other frequently affected areas include the knees, midtarsal joints, and tendinous structures such as the quadriceps and patellar tendons [4].

In contrast, axial gout has been considered a rare entity. However, emerging evidence suggests that spinal involvement may be under-recognised, especially in individuals with chronic or poorly controlled disease. Studies have reported varying estimates of axial gout prevalence. Jin et al. found that 15.8% of Korean patients with spinal symptoms had axial gout, with the lumbar spine being the most commonly affected region [5]. Similarly, Konatalapalli et al. reported that 35% of patients with poorly controlled gout had CT evidence of spinal erosions and/or tophi, with involvement seen predominantly in the lumbar spine, followed by the cervical spine and sacroiliac joints [6]. Specific to facet joint involvement, de Mello et al. found that 17% of patients with axial gout had interapophyseal joint erosions or calcifications [7].

Risk factors for developing spinal gout include hyperuricemia, chronic gout duration, and comorbidities such as obesity, hypertension, diabetes, and chronic kidney disease [8].

Clinically, facet joint gouty arthropathy typically presents with chronic low back pain. Complications-wise, tophi in the lumbar spine can compress nerve roots, leading to radiculopathy. This manifests as severe lower back pain, radiating pain to the extremities, and neurological deficits such as weakness or numbness in the limbs [8]. Chronic compression of the spinal cord by tophi can lead to myelopathy. Gouty tophi can also invade the vertebral joints and protrude into the spinal canal, causing spinal canal stenosis, which could potentially result in irreparable spinal cord injury if not diagnosed and treated early. Erosive changes in the facet joints due to tophi can lead to joint instability and chronic pain. This can further exacerbate spinal deformities and functional impairment. The presence of large tophi can weaken the vertebral structure, predisposing patients to pathological fractures, which can complicate the clinical course and require surgical intervention [9]. Spinal gout can have severe consequences that can significantly impair mobility, functionality, and quality of life. Early recognition and appropriate management - including pharmacological therapy, lifestyle modifications, and, in severe cases, surgical intervention - are crucial to prevent irreversible complications. This will be further discussed in subsequent sections.

However, spinal gout remains underdiagnosed due to its rarity and nonspecific symptoms, which can mimic other spinal pathologies. The differential diagnosis of chronic lumbar pain is broad and includes degenerative facet osteoarthritis, discogenic pain, spondyloarthropathies, infection, neoplasms, and other crystal arthropathies [10]. Inflammatory features on imaging (e.g., facet joint effusion, bone marrow oedema) are not specific, and routine MRI reports may underrecognize these findings [11]. A high index of suspicion is therefore essential in patients with poorly controlled gout who present with chronic spinal pain. Further investigations recommended for distinguishing gout from other aetiologies will be discussed in subsequent sections.

## Case presentation

A 27-year-old Malay gentleman presented to the Sport and Exercise Medicine clinic with left-sided lower back pain radiating to the left thigh for the last six months. He reported intermittent lower back pain for the last three years, with symptoms worsening over the last six months. This happened insidiously without any precipitating trauma. There was lower back stiffness with no associated paraesthesia, numbness, or weakness. He reported that his symptoms worsened with activities involving both flexion and extension, and were aggravated by prolonged sitting, standing, and lying down. 

On examination, the patient exhibited tenderness over the L4/5 region, with a globally limited lumbar range of motion due to pain and a large body habitus. The straight leg raise test demonstrated tight hamstrings bilaterally at 60 degrees, with no sciatica. Neurological examination of the lower limb, including tone, power, sensation and reflexes, was intact. Notably, he had gouty tophi with tenderness and swelling over his right elbow and right lateral malleolus.

The patient had a background of morbid obesity (BMI > 40) and poorly controlled gout. His gout was first diagnosed at age 17 when he presented with episodic bilateral foot and ankle pain. Subsequently, he underwent right ankle arthroscopic debridement and os trigonum excision, where intra-operative findings revealed gout-like deposits over the posterior talus. Histopathological examination confirmed amorphous uric acid deposits, and his serum uric acid level was 707 μmol/L (Table 1). He was subsequently started on allopurinol but had been non-compliant with treatment and defaulted on follow-ups.

**Table 1:** Lab Results

Lab results	Baseline result (done 10 years ago, at 17 years old)	Latest result (done at 27-years-old, before re-initiating treatment)	Normal reference range
Uric acid level (serum)	707 µmol/L	594 µmol/L	218-578 µmol/L

**Figure 1:**
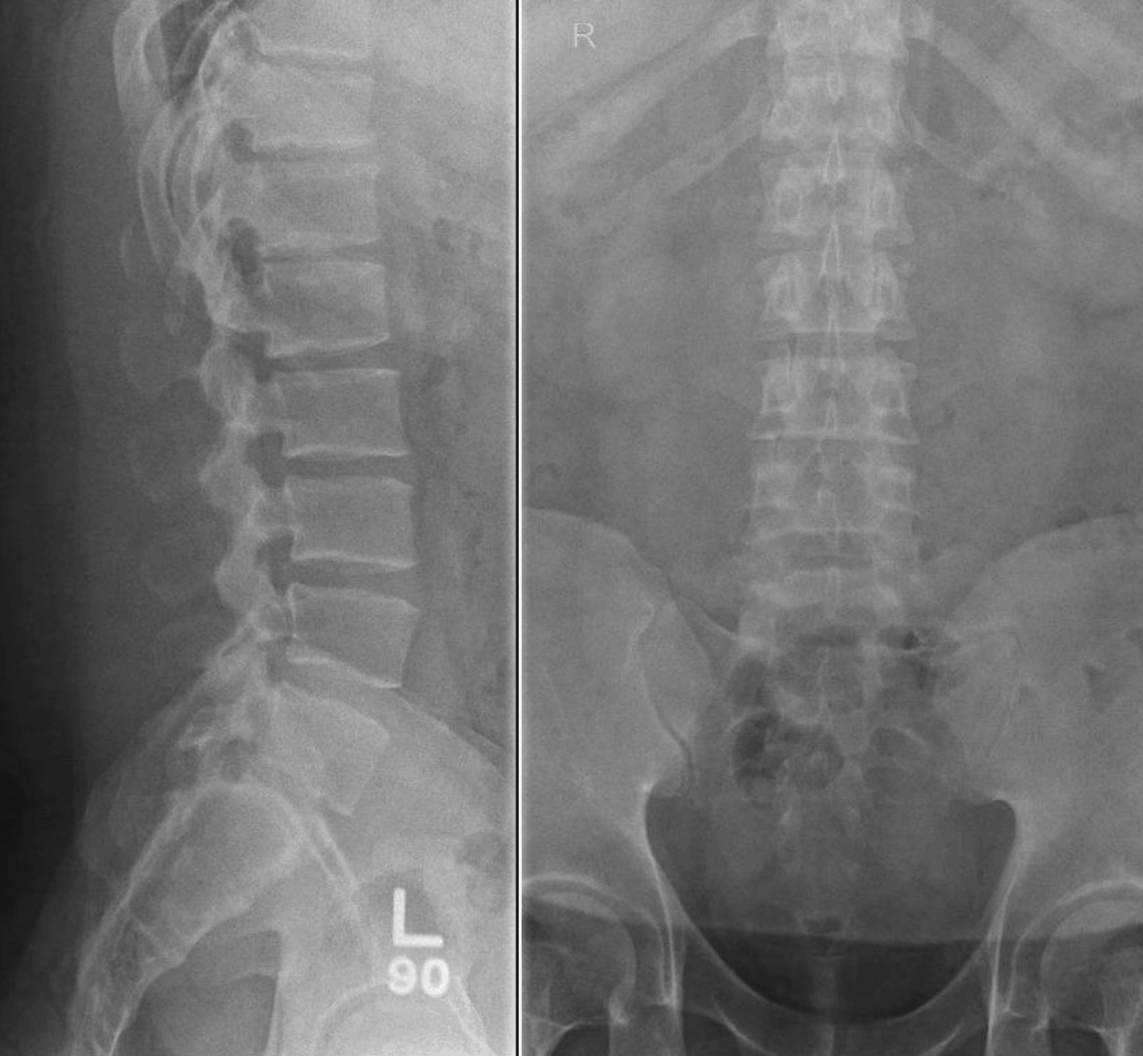
Lumbar Spine X-ray

**Figure 2:**
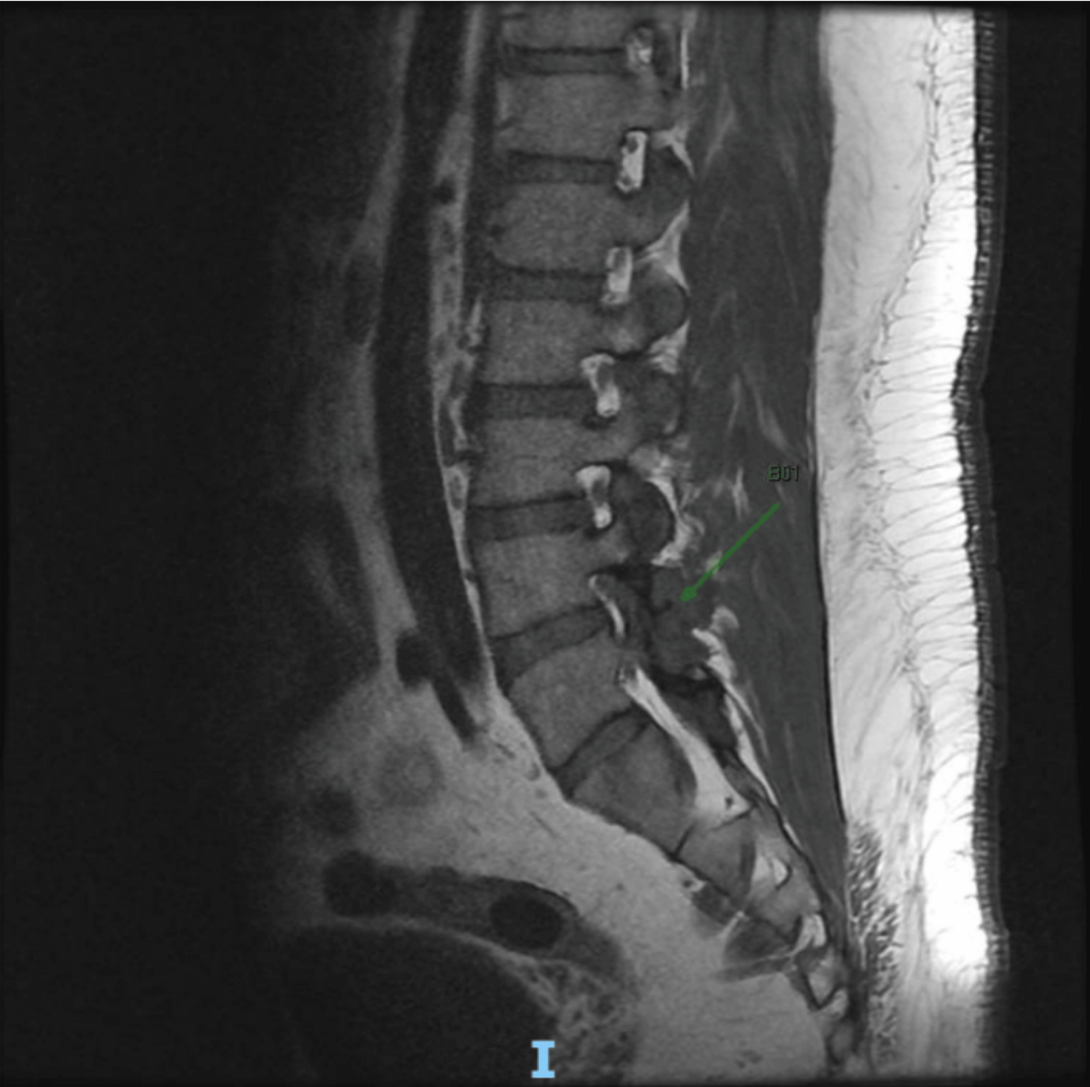
MRI Lumbar Spine Sagittal View, T1-Weighted

**Figure 3:**
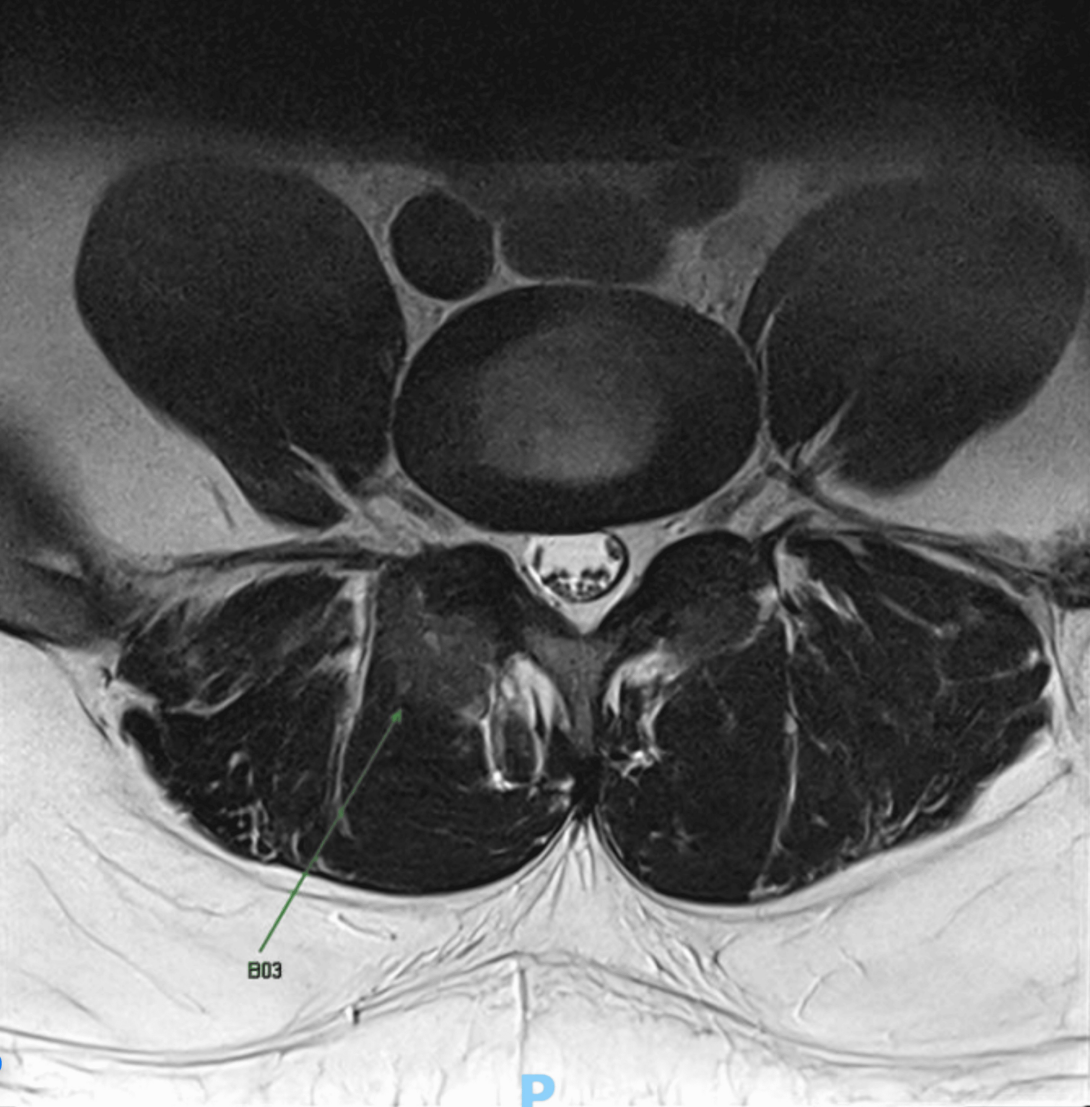
Contrast-Enhanced MRI Lumbar Spine Axial View, T2-Weighted

The patient was referred for a lumbar spine X-ray, which showed mild loss of lumbar lordosis and preserved vertebral body heights and normal intervertebral disc space (Figure 1). A contrast-enhanced MRI of the lumbar spine was done, revealing T1-weighted contrast enhancement at the L4-L5 facet joint area and a possible punched-out erosion (Figures 2-3), suggestive of facet joint gouty arthropathy and tophaceous changes, given his history of poorly controlled gout.

A repeat uric acid level was done, which was 594 μmol/L. His allopurinol was subsequently restarted upon diagnosis of axial gout and uptitrated to 400 mg following consultation with the rheumatologist. He was also given etoricoxib and oral prednisolone for gout flares, as well as a prophylactic dose of colchicine upon starting treatment. He was also referred to a dietitian and started on a weight management programme for his obesity.

He is still undergoing treatment and follow-up with rheumatology and is pending repeat uric acid levels and monitoring for improvement of symptoms.

## Discussion

This case underscores the potential for spinal involvement in gout, emphasizing the need for early and sustained urate-lowering therapy (ULT) to prevent atypical manifestations of gout arthropathy. Spinal gout should be considered as a differential diagnosis when patients present with lower back pain and have risk factors such as chronic gout, obesity and male gender. In this case, the presence of these risk factors, including a long-standing history of poorly controlled gout (≥10 years), obesity, and male gender, is a key contributor to gout pathogenesis.

The prevalence of spinal gout in young adults aged 20 to 30 years is currently not very well quantified in the medical literature. Most published cases occur in older adults, typically between ages 45 and 80 years, with systematic reviews indicating that the mean age of spinal gout is approximately 58 years. Case reports in adults 30 or under are exceptional and typically associated with longstanding, severe, or early-onset gout, often with underlying risk factors such as renal dysfunction or genetic predisposition. They are mainly symptomatic with symptoms, such as axial pain, radiculopathy, or neurological deficits, which prompt further investigation and ultimately lead to diagnosis [12].

In clinical practice, axial gout is frequently underdiagnosed as its symptoms overlap with more common spinal pathologies like disc herniation, degeneration and spinal stenosis. Diagnosing spinal gout often requires a combination of imaging and histopathological confirmation. MRI findings of spinal gouty tophi typically manifest as homogeneous intermediate to low T1 signals with variable T2 intensities. Gadolinium enhancement suggests vascularized tissue within the tophus. Although histopathological confirmation via image-guided fine needle aspiration or biopsy remains the gold standard, it is invasive and carries procedural risks [9].

A promising non-invasive diagnostic tool, dual-energy computed tomography (DECT), can detect urate deposits and potentially reduce the need for invasive procedures. DECT has demonstrated high sensitivity (84.7%) and specificity (93.7%) for detecting monosodium urate (MSU) deposits, making it a reliable diagnostic tool [13]. Additionally, DECT allows for quantification of urate burden, aiding in treatment monitoring, and can detect subclinical MSU deposits that may not be evident on conventional imaging [14]. However, limitations exist: DECT is costlier than conventional imaging, has lower sensitivity in early-stage disease (potentially leading to false negatives), and is susceptible to artifacts requiring advanced post-processing algorithms [15].

Despite growing recognition of spinal gout, significant gaps remain in its diagnosis, management, and long-term outcomes. As discussed, spinal gout is underreported and underdiagnosed due to its rarity and nonspecific symptoms, which often mimic more common spinal pathologies. In Sport and Exercise Medicine clinics, where axial symptoms are frequently attributed to degenerative spinal disease or disc prolapse, spinal gout may be overlooked, leading to delayed or missed diagnoses. A major challenge is the lack of long-term outcome studies. Most available data are limited to case reports and small case series, leaving uncertainties regarding the natural history, disease progression, and optimal treatment strategies for spinal gout. Without robust longitudinal studies, clinicians must rely on extrapolated data from peripheral gout, which may not fully apply to axial involvement.

Management of axial gout involves acute symptom relief, as well as long-term ULT to prevent recurrence. Treatment of an acute gout flare involving the facet joint mirrors that of peripheral gout. First-line non-steroidal anti-inflammatory (NSAIDs) may be used if there are no contraindications for a short course. Colchicine can also be used for acute gout flares as well as prophylactically at a low dose (0.6 mg once or twice daily) to prevent flares when starting ULT. Oral corticosteroids may also be used in acute gout flares, such as oral prednisolone, for short courses. Intra-articular corticosteroid management may be considered if the diagnosis is confirmed and the facet joint can be accessed, usually done under fluoroscopy or CT guidance. Systemic steroids may be necessary for those with polyarticular involvement or severe flares [12].

Chronic management involves using ULT. The indications to start ULT normally include two or more gout attacks per year, tophi, chronic gouty arthritis, radiographic changes, renal stones and spinal involvement, like facet joint disease. First line ULT is normally xanthine oxidase inhibitors such as allopurinol. Febuxostat may be an alternative for those with allopurinol intolerance, but it should be used with caution in those with cardiovascular disease. Second-line uricosuric agents like probenecid may be used if xanthine oxidase inhibitors are contraindicated or not tolerated [16].

Lifestyle and risk factor management play a crucial role in the management of both axial and peripheral gout. Patients should reduce alcohol intake, purine-rich foods, and fructose. Obesity, hypertension, metabolic syndrome and renal function should also be managed accordingly. Hydration should always be encouraged in patients with gout [17].

Given the complexity of spinal gout, a multidisciplinary approach is essential with collaboration between family physicians, sports medicine specialists, pain team and rheumatologists, to ensure comprehensive evaluation, appropriate imaging selection, and personalised management. Greater awareness among clinicians across specialties is also crucial, as recognising atypical gout presentations can facilitate earlier diagnosis and intervention, ultimately improving patient outcomes.

## Conclusions

This case highlights an atypical presentation of gouty arthropathy affecting the lumbar facet joints, underscoring the importance of maintaining a high level of clinical suspicion in patients with longstanding gout who present with ongoing back pain. From a sports medicine perspective, this case reinforces the importance of recognizing systemic conditions like gout, which can present atypically and contribute to musculoskeletal (MSK) dysfunction. Axial pain in active individuals is often attributed to mechanical or degenerative causes, potentially delaying the diagnosis of metabolic arthropathies such as gout.

Optimized ULT and anti-inflammatory treatment remain the cornerstone of management, but treatment plans must be tailored to address the MSK consequences. In addition to pharmacologic intervention, rehabilitation strategies focusing on pain management and mobility restoration are critical for functional recovery. Recognizing and managing comorbidities such as obesity and metabolic syndrome is essential in preventing disease progression and improving overall patient outcomes. A multidisciplinary approach involving rheumatologists, family physicians, sports medicine specialists, and rehabilitation teams is crucial to ensuring a comprehensive, patient-centred treatment plan. Early recognition, targeted interventions, and coordinated care can enhance both systemic disease control and functional recovery, ultimately improving the patient’s quality of life.
